# Maintenance of time-restricted eating and high-intensity interval training in women with overweight/obesity 2 years after a randomized controlled trial

**DOI:** 10.1038/s41598-025-95743-y

**Published:** 2025-04-25

**Authors:** Kamilla L. Haganes, John A. Hawley, Stian Lydersen, Trine Moholdt

**Affiliations:** 1 Department of Circulation and Medical Imaging, Faculty of Medicine and Health Sciences, Norwegian University of Science and Technology, Trondheim, 8905, 7491 Norway; 2 Women’s Clinic, St. Olav’s Hospital, Trondheim, 7006 Norway; 3 Exercise & Nutrition Research Program, the Mary Mackillop Institute for Health Research, Australian Catholic University, Melbourne, VIC 3000 Australia; 4 Department of Sport and Exercise Sciences, Manchester Metropolitan University Institute of Sport, Manchester, UK; 5 Department of Mental Health, Faculty of Medicine and Health Sciences, Norwegian University of Science and Technology, Trondheim, 7491 Norway

**Keywords:** Long-term adherence, High-intensity interval training, Obesity, Overweight, Time-restricted eating, Cardiorespiratory fitness, Lifestyle interventions, Physical activity, Diet, Cardiology, Nutrition, Public health, Weight management, Clinical trial design, Translational research, Cardiovascular diseases, Metabolic disorders, Lifestyle modification

## Abstract

Time-restricted eating (TRE) and high-intensity interval training (HIIT) improve cardiometabolic health in individuals with overweight/obesity, with high adherence rates in supervised settings. Long-term maintenance of TRE and HIIT in real-world settings is unknown. In our previous TREHIIT trial, 131 women (body mass index (BMI) ≥ 27 kg/m^2^) were randomized to 7 weeks of TRE (eating window 10-h/day), HIIT (3 sessions/week), a combination (TREHIIT), or no intervention (CON). We investigated self-reported continuation of TRE and/or HIIT after 2 years. Fifty-nine participants (39.0 years (standard deviation (SD) 6.1), BMI 30.7 kg/m^2^ (SD 4.2)) attended the follow-up. Of those who completed the 7-week TRE or HIIT intervention, 46% maintained TRE and 45% continued HIIT for 2 years. There were no statistically significant (at *p* < .01) between-group differences in cardiometabolic outcomes, but non-significant lower body mass in HIIT (-4.2 kg, 95% confidence interval (CI), -7.7 to -0.7, *p* = .019) and visceral fat in TREHIIT (-18 cm^2^, CI, -33 to -4, *p* = .015) versus CON. After 2 years, HIIT and TREHIIT had ~ 4 kg lower fat mass and ~ 20 cm² lower visceral fat (both *p* < .001) compared with baseline. A short-term TRE and HIIT intervention may promote long-term lifestyle changes and health benefits. Future studies should collect objective adherence data to understand long-term maintenance of TRE and HIIT.

**Supplementary Information:**

The online version contains supplementary material available at 10.1038/s41598-025-95743-y.

## Background

Dietary restriction and increased physical activity are primary lifestyle interventions to prevent and manage overweight/obesity and associated conditions. However, many individuals who initially lose weight after diet-exercise interventions experience poor long-term outcomes, largely due to challenges in maintaining healthy lifestyle changes^1–3^. Continuous energy restriction is often unsustainable in the long-term due to factors such as the need for constant self-monitoring of energy intake and avoidance of specific foods^1,4,5^. While low-energy diets promote initial weight loss, weight regain after one year is common^3,6,7^.

In recent years, time-restricted eating (TRE) has emerged as a popular and practical dietary strategy that may be easier to sustain than continuous energy restriction (CER) or intermittent fasting (IF) protocols. TRE plays off normal circadian rhythm and energy metabolism by limiting daily energy intake to a 6–12-hour window, which improves numerous measures of metabolic and cardiorespiratory health^8–11^. Indeed, CER and other IF protocols are not chrono-nutritive therapies per se, in that they do not restrict food consumption to between specified times of day to play off of chronobiology^12^. Although there are no constraints on energy intake or dietary composition, TRE often leads to spontaneous reductions in energy intake, inducing moderate weight loss (3–5%) over several months^10^. Key advantages of TRE, such as freedom from constant energy monitoring and allowing personal dietary choices, may overcome some of the challenges associated with traditional energy restrictive diets^5,10^. Environmental factors, availability of highly palatable foods, and increased feelings of hunger are identified as common predictors of lapses from several restrictive diets^13^. Therefore, being able to consume preferred foods within the TRE window may promote long-term adherence to TRE^14,15^. In supervised settings and study durations of up to 16 weeks, adherence to TRE is consistently high, with adherence rates of around 90%^16^. Even though several studies report high interest among participants to continue TRE beyond the active intervention period, there are limited long-term, real-world data on TRE adherence^16^.

Higher levels of physical activity are associated with successful weight maintenance after diet-induced weight loss^7,17–20^. High-intensity interval training (HIIT), consisting of repeated short bouts of intense endurance exercise interspersed with low-intensity recovery periods, induce similar changes in body composition as moderate-intensity continuous exercise, which is traditionally recommended for weight maintenance^21^. HIIT has gained popularity due to its time efficiency and effectiveness in improving cardiorespiratory fitness^22–24^, a strong independent risk factor for type 2 diabetes and cardiovascular disease^19,20^. A recent systematic review found that inactive adults, both with and without medical conditions, had high adherence rates to HIIT interventions in supervised settings, with ~ 90% of sessions completed^25^. However, in unsupervised, real-world settings, adherence to HIIT dropped to 63%, similar to the reported adherence rates of 68% for moderate-intensity continuous training^25^.

In the randomized controlled TREHIIT trial, we allocated women with a body mass index (BMI) ≥ 27 kg/m^2^ to either TRE, HIIT, a combination (TREHIIT), or a no-intervention control group (CON)^26^. Seven weeks of TRE and HIIT independently reduced fat mass and visceral fat area, while the combination of TRE and HIIT induced greater reductions than either intervention alone^27^. Adherence to both TRE and HIIT was excellent during the 7-week intervention period, with participants completing ~ 95% of scheduled HIIT sessions and adhering to the ≤ 10-h TRE window on 6.1 (standard deviation (SD) 1.0) days/week^27^. Despite consistent reports of beneficial short-term effects and high adherence to TRE and HIIT under controlled, supervised conditions^16,25^, sustained behavioural change can be difficult^2^. Free-living conditions present a dynamic context in which people need flexible and practical lifestyle interventions to facilitate long-term adherence. To our knowledge, there is limited evidence for long-term maintenance of TRE and HIIT in women with overweight/obesity. In this follow-up study, we aimed to examine self-reported continuation of TRE and HIIT, and health outcomes, 2 years after the participants completed the supervised 7-week intervention in the TREHIIT trial, without any instructions on continuation beyond the intervention period.

## Methods

### Study population

In the TREHIIT trial (Clinicaltrials.gov NCT04019860), women aged 18–45 years with a BMI ≥ 27 kg/m^2^ (*N* = 131) were randomly allocated (1:1:1:1) to 7 weeks of either TRE (*n* = 33), HIIT (*n* = 33), TRE and HIIT (TREHIIT, *n* = 32), or no intervention (CON, *n *= 33) using a random number generator (The Unit for Applied Clinical Research, NTNU, Trondheim). Participants and study investigators were not blinded for group allocation. We have published detailed information about the trial methods and primary results^26,27^. All participants who completed post-assessments after the 7-week intervention and who consented to future contact (*n* = 106) were eligible for the follow-up study and were contacted by email or telephone 2 years after randomization in the TREHIIT trial. The participants were not aware of the follow-up study prior to being contacted after 2 years. The follow-up study was approved by the Regional Committee for Medical and Health Research Ethics in Middle Norway (REK no. 285171) and conducted in accordance with the Declaration of Helsinki. All participants provided informed consent prior to the assessments.

### Interventions

There were three intervention groups (TRE, HIIT, and TREHIIT) and one control group (CON) in the TREHIIT trial. Detailed descriptions of the interventions can be found in the original article^27^. We instructed participants allocated to TRE to limit their energy intake to a self-selected ≤ 10-h daily eating window, finishing no later than 20:00 h, without any advice on total energy intake or food composition. The participants were allowed non-energy-containing beverages during the fasting period. Participants allocated to HIIT performed three weekly supervised exercise sessions at the laboratories at St. Olav’s Hospital as treadmill running or stationary bicycling. Study investigators with background in exercise physiology supervised the exercise sessions. During the COVID-19 lockdown from March to August 2020, the HIIT sessions were performed as outdoor running or walking. The exercise programme consisted of two weekly 4 × 4-min HIIT sessions, with work-bouts performed at 90–95% of maximal heart rate, and one weekly 10 × 1-min HIIT session, with work-bouts performed at the maximum intensity the participants could sustain for 1 min. Between each work-bout in the 4 × 4-min HIIT sessions, participants had a 3-min low-to-moderate intensity active recovery period, while participants could choose to stand still or walk at low intensity during the 1-min recovery periods in the 10 × 1-min HIIT sessions. Participants in the TREHIIT group followed both TRE and HIIT. Participants in CON received no intervention for 7 weeks but were offered to choose one of the study interventions as a delayed treatment after completing post-assessments. We did not give any advice to continue the assigned interventions beyond the intervention period. The TIDieR (Template for Intervention Description and Replication) checklist and CONSORT checklists are provided in Supplementary file 3^28,29^.

### Laboratory assessments

The laboratory assessments in the follow-up study were identical to that of the TREHIIT trial^27^, except we did not perform a 2-h 75-g oral glucose tolerance test in the follow-up study. We instructed participants to abstain from vigorous physical activity for ≥ 48 h prior to the measurements and to fast from ≤ 22:00 h the night before attending the laboratory. The assessments were undertaken at St. Olav’s hospital’s laboratories and scheduled to the follicular phase for women with a regular menstrual cycle. We collected fasting blood samples and analysed concentrations of fasting plasma glucose, total cholesterol, triglycerides, high-density lipoprotein (HDL) cholesterol, low-density lipoprotein (LDL) cholesterol, and glycated haemoglobin (HbA1c), as previously described^26,27^. The St. Olav’s laboratories used a homogeneous assay for directly measuring LDL cholesterol (Siemens Atellica CH930), and high-performance liquid chromatography (Tosoh G8LA Variant mode) to measure HbA1c. We aliquoted and stored additional serum, plasma and fullblood at −80 °C in a biobank for later analyses. We used bioelectrical impedance analysis (InBody720, Biospace CO, Korea) to estimate body composition in the morning after the ≥ 10-h overnight fast. We measured blood pressure and resting heart rate (Philips IntelliVue MP50, Philips Medizin Systeme, Germany) while participants were in a rested, seated position. We report the average of three consecutive measurements taken 1 min apart. We measured peak oxygen uptake (VO_2_peak) with indirect calorimetry (MetaMax II Portable CPX System, Cortex, Germany) during a cardiorespiratory fitness test following an individualized ramp protocol on a treadmill, as described and recommended by the American College of Sports Medicine^26,27,30^. The participants completed The International Physical Activity Questionnaire^31^, the Pittsburgh Sleep Quality Index^32^, and an internally-developed questionnaire assessing long-term continuation and perceptions of the interventions employed in the TREHIIT trial (Supplementary file). The latter questionnaire examined maintenance of TRE and/or HIIT after intervention completion, and potential drivers or barriers to TRE and/or HIIT. Participants rated their perceptions of TRE and/or HIIT compared with other diet and exercise strategies they had previously tried on a 10-point Likert scale (values ranging from 1 to 10, where 1 = “much worse”, 5 = “neither better nor worse”, and 10 = “much better”).

### Outcome measures

The primary outcome was the number of participants who reported adopting TRE and/or HIIT, 2 years after completing a 7-week TRE and/or HIIT intervention in the TREHIIT trial. For TRE, we report the proportion of participants who reported still following a TRE pattern after 2 years and the number of days/week that participants reported having a ≤ 10-h eating window. For HIIT, we report the proportion of participants who reported undertaking HIIT after 2 years and the average number of HIIT sessions/week. Secondary outcomes include fasting glucose, HbA1c, total cholesterol, HDL- and LDL-cholesterol, triglycerides, systolic and diastolic blood pressure, resting heart rate, total body mass, fat mass, visceral fat area, muscle mass, VO_2_peak, self-reported physical activity levels, and sleep quality.

### Statistical analysis

We did not perform a separate sample size-calculation for this follow-up study. The sample size for the original TREHIIT trial was calculated as described previously^27^, in which 24 participants in each group were needed to detect a difference of −54 (64) mmol/L in total area under the glucose curve between the HIIT and CON group, using statistical power of 80% and significance level α = 0.05 for a 2-sided, independent t-test. In the analyses, we included data from all participants in the TREHIIT trial, regardless of outcome measure completeness (intention-to-treat). Participants were analysed according to the initial group they were allocated to. Long-term intervention continuation data are reported as descriptive statistics. We used linear mixed models with time and the interaction between time and group (time x group) as fixed effects, and participant as random effect to investigate between-group differences in cardiometabolic outcomes after 2 years and within-group differences after 2 years compared with baseline and compared with post-intervention. The time variable was categorized as baseline, 8 weeks (after the intervention period), and 2 years. We adjusted for baseline values, assuming no systematic effect of group at baseline, as recommended by Twisk et al.^33^. In the primary analyses, estimated intervention effects are mean changes over time in the intervention groups compared with CON. In the secondary analyses, within-group differences were estimated as mean changes between the 2-year follow-up versus baseline and versus post-intervention (8 weeks). We inspected normality of residuals by visually checking QQ-plots. Due to multiple comparisons, we consider 2-sided *p* values < 0.01 as statistically significant. We performed all statistical analyses in IBM SPSS Statistics 27 and generated figures in Microsoft^®^ Word Version 2312, Microsoft^®^ PowerPoint Version 2403, and GraphPad Prism 9.

## Results

### Participants

**Fig. 1:**
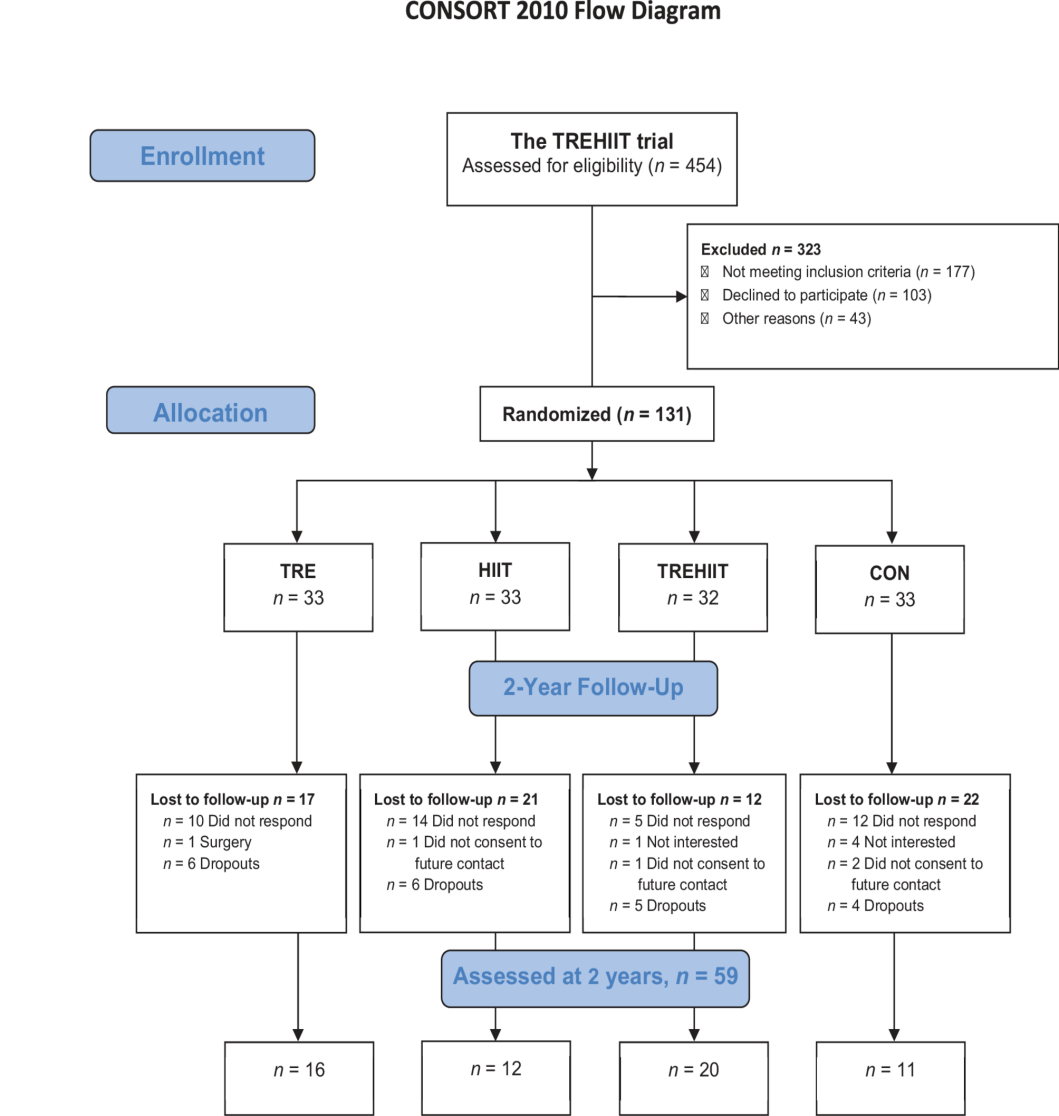
Flow chart of participants *CON* control group, *HIIT* high-intensity interval training, *TRE* time-restricted eating, *TREHIIT* time-restricted eating and high-intensity interval training.

Of those eligible (*N* = 106), 59 (56%) consented to partake in the follow-up study and were assessed between October 2021 and March 2023, which was 24.4 (1.2) months after completing the original trial (Fig. 1). Participants were 39.0 (6.1) years and had a BMI 30.7 (4.2) kg/m^2^ at the 2-year follow-up. Baseline characteristics were similar for the participants who attended the 2-year follow-up and those who declined, did not respond, or dropped out of the TREHIIT trial (Table 1). One participant in TREHIIT and one participant in CON completed questionnaires but did not attend laboratory assessments. One participant in TRE had started taking Semaglutide for weight loss and was excluded from the analyses of cardiometabolic outcomes at 2 years. Eight of the CON participants had chosen delayed intervention after completing the control period in the TREHIIT trial: five selected the HIIT intervention and three selected the combined intervention (TREHIIT).

**Table 1:** Descriptive baseline characteristics for participants and non-participants in the 2-year follow-up study.

		2-year follow up participants (*n* = 59)		Non-participants (*n* = 72)
	*n*	**Mean (SD)**	*n*	**Mean (SD)**
**Age**, years*	59	36.9 (6.0)	*72*	35.7 (6.3)
**Body weight**,** kg**	59	89.3 (11.6)	72	93.2 (11.2)
**BMI**,** kg/m**^**2**^	59	31.9 (4.2)	72	32.5 (3.8)
**Muscle mass**,** kg**	59	29.2 (2.7)	72	30.4 (2.8)
**Fat mass**,** kg**	59	36.9 (9.5)	72	38.6 (9.0)
**Visceral fat area**,** cm**^**2**^	59	177 (44)	72	185 (42)
**Systolic BP**,** mmHg**	59	122.5 (10.5)	72	122.7 (10.6)
**Diastolic BP**,** mmHg**	59	80.2 (8.6)	72	80.5 (8.4)
**HbA1c**,** mmol/mol**	57	34.1 (3.1)	69	33.9 (3.4)
**Fasting glucose**,** mmol/L**	59	5.0 (0.4)	71	4.9 (0.4)
**Total cholesterol**,** mmol/L**	57	4.8 (0.8)	69	4.7 (0.8)
**Triglycerides**,** mmol/L**	56	1.1 (0.4)	69	1.2 (0.5)
**HDL cholesterol**,** mmol/L**	57	1.4 (0.3)	69	1.3 (0.3)
**LDL cholesterol**,** mmol/L**	57	3.3 (0.8)	69	3.3 (0.9)
**VO**_**2**_**peak**,** L/min**	59	3.1 (0.4)	67	3.2 (0.4)
**VO2peak**,** mL/min/kg**	59	35.1 (5.2)	72	34.4 (5.7)
**IPAQ score**	59	1632 (1868)	58	1806 (2086)
**PSQI score**	57	6.4 (3.5)	59	6.4 (3.6)

*BP* blood pressure, *BMI* body mass index, *HbA1c* glycated haemoglobin, *HDL* high-density lipoprotein, *IPAQ* International physical activity questionnaire, *LDL* low-density lipoprotein, *PSQI* Pittsburgh sleep quality index, *VO*_*2*_*peak* peak oxygen uptake.

## Two-year continuation of TRE and HIIT (Primary outcome)

### Self-reported continuation in the TRE group

**Fig. 2:**
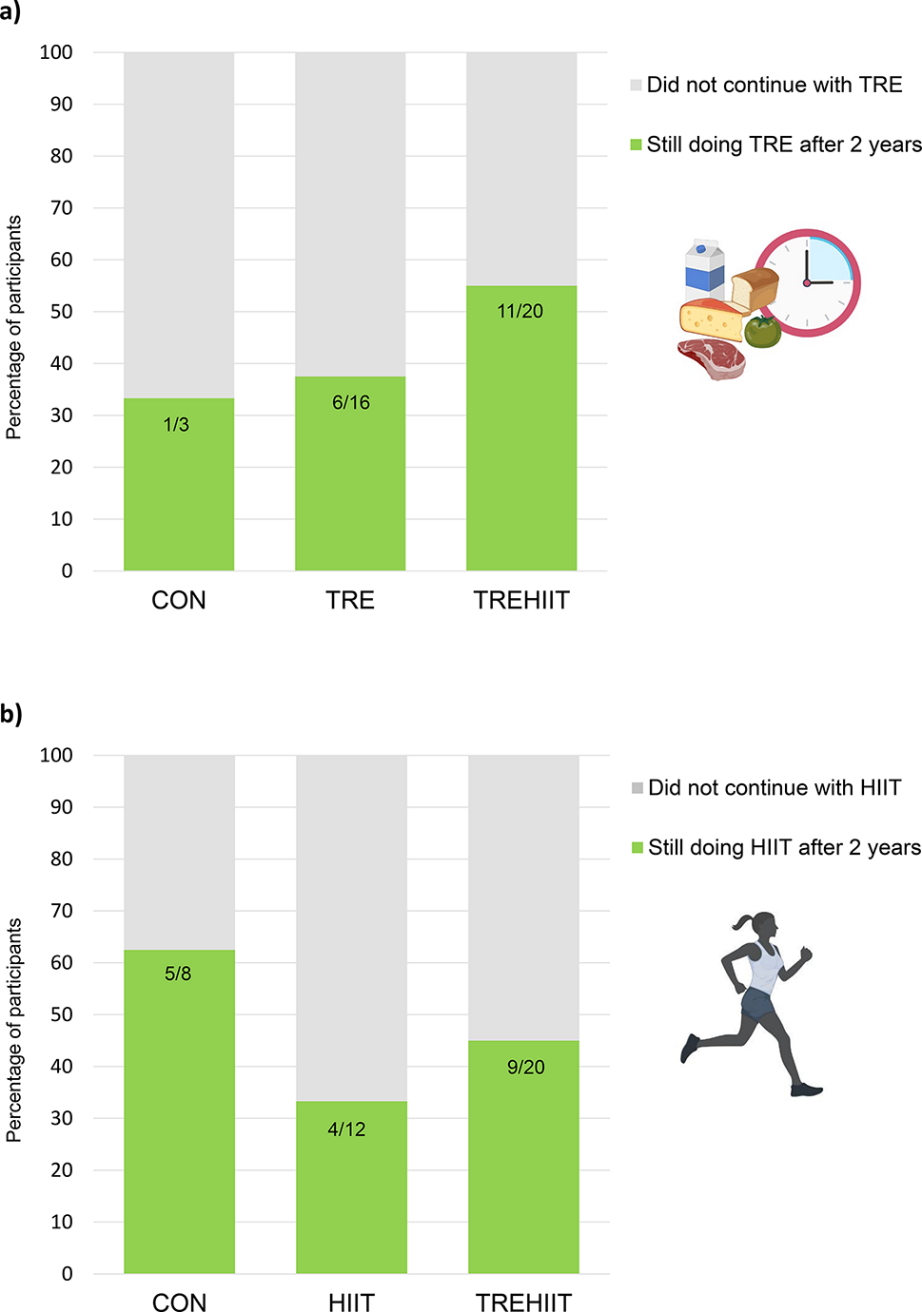
Adherence to time-restricted eating (TRE) and high-intensity interval training (HIIT) 2 years after study participation. **a**) Adherence to TRE according to originally assigned study group, and **b**) adherence to HIIT according to originally assigned study group. *CON* control group, *HIIT* high-intensity interval training, *TRE* time-restricted eating, *TREHIIT* time-restricted eating and high-intensity interval training.

 Of participants originally allocated to TRE, 6/16 (37.5%) still practiced TRE after 2 years and reported a ≤ 10-h eating window on 4.8 (1.3) days/week (Fig. 2). Additionally, 5/16 (31.3%) participants reported that they continued TRE for 31.2 (19.9) weeks after study completion, whereas 5/16 did not continue. Six of the participants who continued with TRE, reported to have a longer eating window duration on the weekend compared with the weekdays. The average eating window duration was 9.4 (1.8) h/day on weekdays and 10.9 (2.0) h/day on weekends. Additionally, 10/16 participants started with one or more forms of exercise training after study completion, including HIIT (*n* = 4), group exercise (*n* = 5), resistance training (*n* = 5), moderate-intensity continuous exercise (*n* = 3), outdoor walking (*n* = 4), or stationary cycling (*n* = 1).

## Self-reported continuation in the HIIT group

In the original HIIT group, 4/12 (33%) participants reported still undertaking HIIT after 2 years, while one continued for 3 weeks and one for 32 weeks (Fig. 2). One participant did not report how long they had continued HIIT. These participants reported completing 1.4 (0.5) HIIT sessions/week. Some of the participants originally allocated to HIIT reported starting with one or more alternative forms of exercise instead of, or in addition to, HIIT, including resistance training (*n* = 4), group exercise (*n* = 2), stationary cycling (*n* = 1), and moderate-intensity continuous exercise (*n* = 2). Additionally, 3/12 reported starting with TRE after study completion.

### Self-reported continuation in the TREHIIT group

In the TREHIIT group, 11/20 (55%) reported still following TRE after 2 years, while 3/20 (15%) followed TRE for 6.3 (2.4) weeks after study completion (Fig. 2). These participants had a ≤ 10-h eating window on 5.9 (1.0) days/week. Six of the participants who continued with TRE had a longer eating window duration on the weekend compared with the weekdays. The average eating window on weekdays and weekends were 9.5 (1.5) h/day and 10.4 (2.2) h/day, respectively. While 6/20 continued with HIIT for some weeks (range 8–56 weeks) after study completion, 9/20 (45%) were still undertaking HIIT after 2 years (Fig. 2) and performed 1.7 (0.8) HIIT sessions/week. Fifteen (75%) participants started with one or more forms of exercise instead of, or in addition to, HIIT, including resistance training (*n* = 8), group exercise (*n* = 3), yoga (*n* = 2), moderate-intensity continuous exercise (*n* = 4), and organized team sports (*n* = 1).

### Self-reported continuation in the CON group

One of the participants in CON reported continuing with TRE for 2 years after the 7-week delayed treatment period, with a 10-h eating window on weekdays and a 12-h eating window on the weekend. Additionally, 5/8 (63%) were still undertaking HIIT 2 years after the delayed treatment, whereas one participant had continued for 8 weeks, and one for 12 weeks (Fig. 2). Those who continued with HIIT completed 1.6 (0.8) sessions/week. In CON, 9/11 (82%) had started with other exercise forms, including resistance training (*n* = 4), moderate-intensity continuous exercise (*n* = 2), yoga (*n* = 1), group exercise (*n* = 1), and organized team sports (*n* = 1).

### Perceptions of TRE and HIIT

**Fig. 3:**
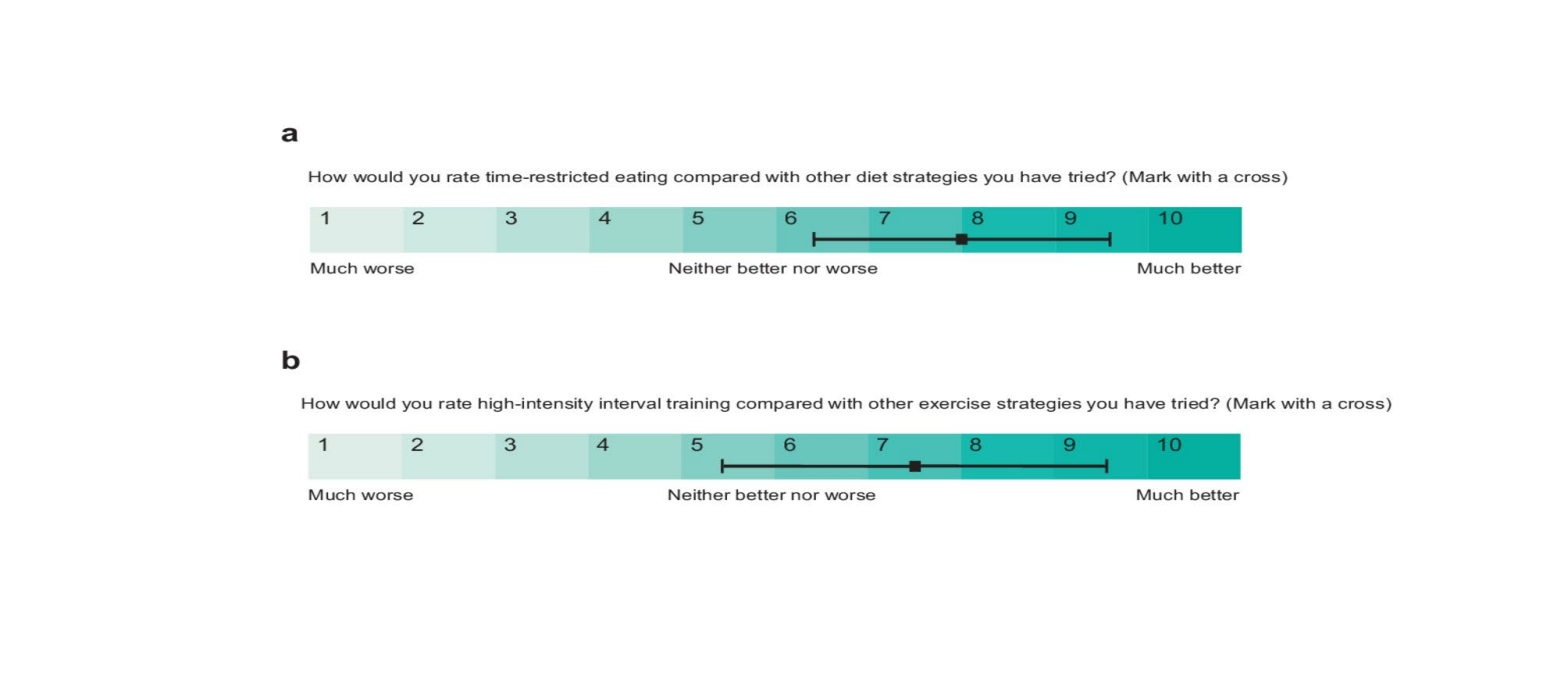
Subjective rating of time-restricted eating (TRE) and high-intensity interval training (HIIT) compared with alternative diet/exercise strategies.

 Compared with other dietary strategies the participants had tried, TRE was rated 8.0 (1.6) points on a 10-point Likert scale (favourable). Compared with other exercise methods, HIIT was rated at 7.5 (2.1) points (Fig. 3). The most common barriers to maintaining TRE were socio-environmental factors, including social events, work-, and family schedules. Other reported barriers to TRE were lack of self-discipline and lack of results. Of the 31 participants in TRE, TREHIIT and CON who provided a qualitative response on TRE perception, 21 reported TRE to be easy to understand and follow. Six participants perceived it as an appealing aspect of TRE to be able to choose what foods to consume (Supplementary Table S1).

**Fig. 4:**
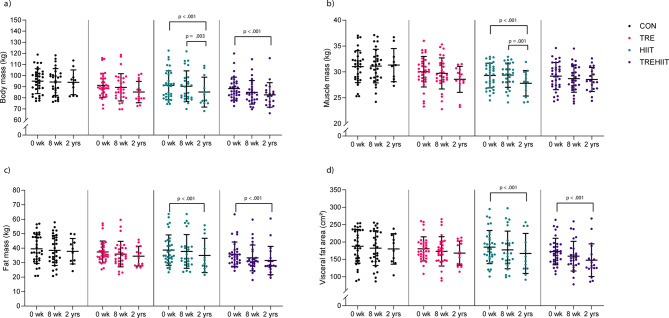
Body composition. Descriptive group means and standard deviations in body composition at baseline (*n* = 131), after the 7-week intervention (*n* = 111), and after 2 years (*n* = 56). **a**) total body mass, **b**) muscle mass, **c**) fat mass, and **d**) visceral fat area. *p*-values are for within-group comparisons. *p* <. 01 are considered statistically significant. *CON* control group, *HIIT* high-intensity interval training, *TRE* time-restricted eating, *TREHIIT* time-restricted eating and high-intensity interval training. Ratings of TRE and HIIT compared with other diet and exercise strategies on a 10-point Likert scale, with means and standard deviations. (a) Mean rating of TRE by the TRE (*n* =14), TREHIIT (*n* = 18), and control (CON, *n* = 3) groups, (b) mean rating of HIIT by the HIIT (*n* = 12), TREHIIT (*n* = 20), and CON groups (*n* = 8). Other diet strategies included: caloric restriction (*n* = 15), low-carbohydrate diet (*n* = 7), commercial weight-loss programs (*n* = 6), 5:2 intermittent fasting (*n* = 3), high-protein diet (*n* = 1). Other exercise strategies included: resistance training (*n* = 16), group exercises (*n* = 6), stationary bicycling (*n* = 1), moderate-intensity continuous training (*n* = 8), yoga (*n* = 3), organized team sports (*n* = 2).

The most stated barrier to performing HIIT was lack of time. Other barriers were injury, pain, and lack of motivation. Three perceived HIIT as too difficult to accomplish and two found the uncomfortable feelings of physical exertion to be a barrier. The most frequently cited positive aspect of HIIT was time-efficiency, followed by enhanced feelings of mastery, proudness, and enjoyment of pushing themselves. Rapid, noticeable improvements in fitness levels were also a motivator for HIIT, while several expressed that they enjoyed HIIT but preferred more variation in their exercise program by including additional exercise modalities (Supplementary Table S1).

### Cardiometabolic outcomes

There were no statistically significant between-group differences in cardiometabolic outcomes after 2 years (Table 2). There were tendencies of greater body mass loss in the HIIT group, a lower absolute VO_2_peak in TRE, and reduced visceral fat area in TREHIIT, compared with CON after 2 years (Table 2).

**Fig. 5:**
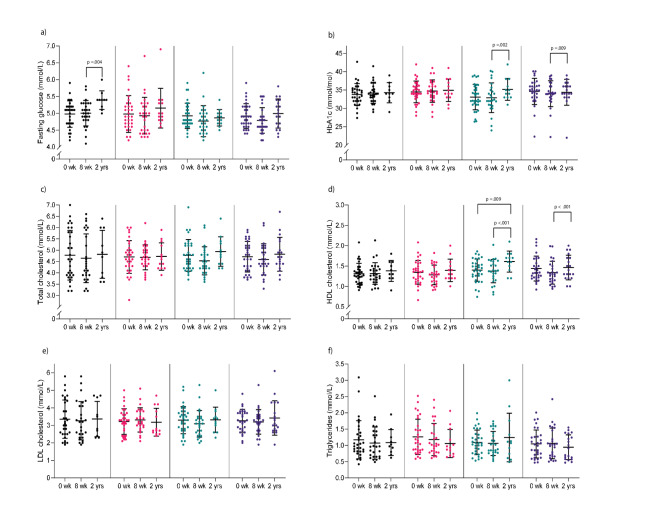
Circulating cardiometabolic outcomes. Descriptive group means and standard deviations in outcomes from fasting blood samples at baseline (*n* = 130), after the 7-week intervention (*n* = 109), and after 2 years (*n* = 54). (a) Fasting glucose, (b) HbA1c, (c) total cholesterol, (d) HDL cholesterol, (e) LDL cholesterol, (f) triglycerides. *p*-values are for within-group comparisons. *p* <. 01 are considered statistically significant. *CON* control group, *HbA1c* glycated haemoglobin, *HDL* high-density lipoprotein, *HIIT* high-intensity interval training, *LDL* low-density lipoprotein, *TRE* time-restricted eating, *TREHIIT* time-restricted eating and high-intensity interval training.

**Fig. 6:**
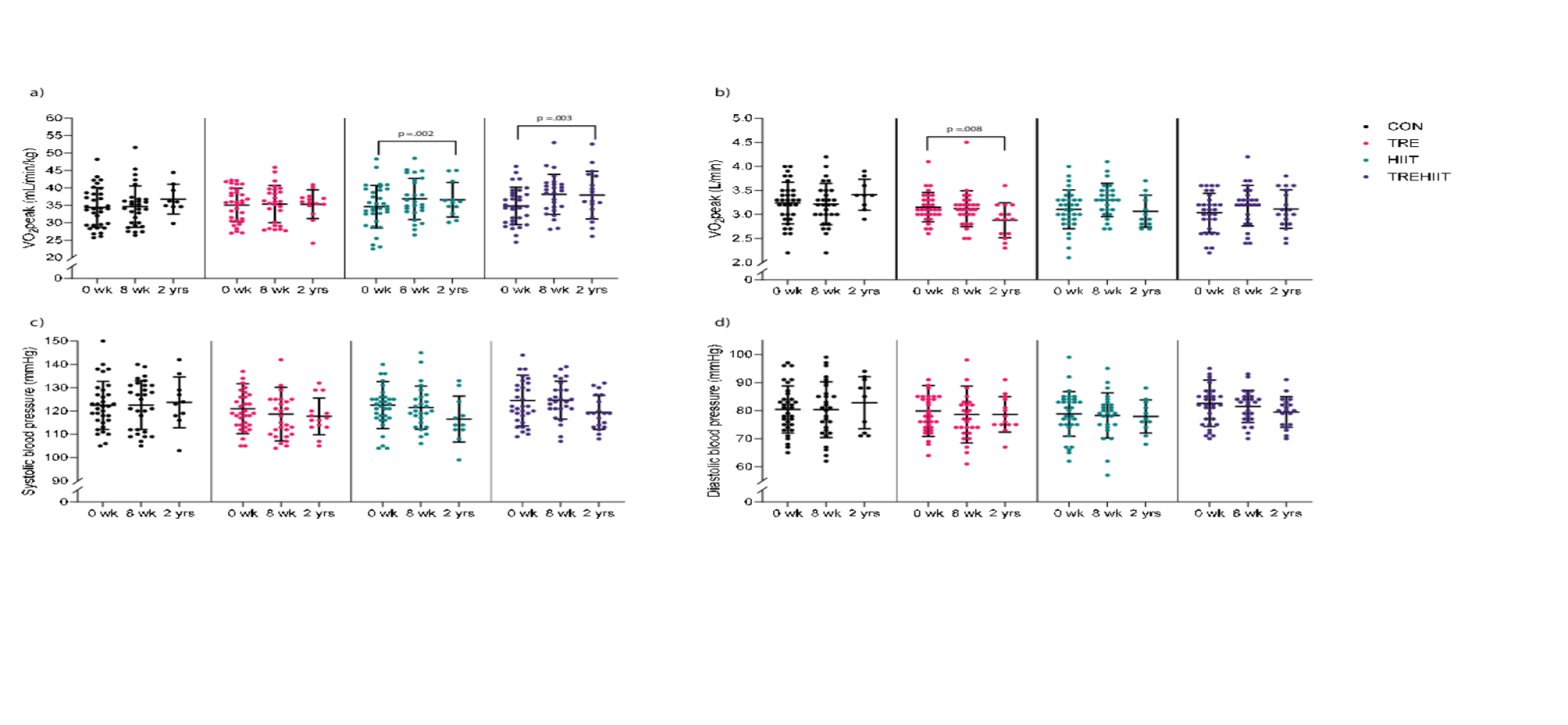
Cardiovascular outcomes. Descriptive group means and standard deviations in peak oxygen uptake and blood pressure at baseline (*n* = 126), after the 7-week intervention (*n* = 108), and after 2 years (*n* = 53). (a) Peak oxygen uptake per kg body weight, (b) absolute peak oxygen uptake, (c) systolic blood pressure, (d) diastolic blood pressure. *CON* control group, *HIIT* high-intensity interval training, *TRE* time-restricted eating, *TREHIIT* time-restricted eating and high-intensity interval training, *VO*_*2*_*peak* peak oxygen uptake.

 In the within-group analyses, participants in HIIT and TREHIIT had significantly lower body mass, fat mass, and visceral fat area after 2 years compared with baseline (Supplementary Table S2, Fig. 4). Specifically, HIIT reduced body mass by 5.3 kg (95%CI −7.7 to −3.0, *p* < .001), fat mass by 4.2 kg (95%CI −6.2 to −2.1, *p* < .001), and visceral fat area by 19 cm^2^ (95%CI, −30 to −8, *p* < .001). TREHIIT reduced body mass by 3.8 kg (95%CI −5.8 to −1.9, *p* < .001), fat mass by 3.9 kg (95%CI −5.6 to −2.2, *p* < .001), and visceral fat area by 22 cm^2^ (95%CI −30 to −13, *p* < .001). In HIIT, body mass was also lower after 2 years compared with after the 7-week intervention period. Participants in HIIT had ~ 0.8 kg lower muscle mass after 2 years compared with baseline and compared with after the 7-week intervention period. Compared with post-intervention, HbA1c and HDL-cholesterol increased after 2 years in HIIT (1.3 mmol/mol, 95%CI 0.5 to 2.1, *p* = .002, and 0.2, 95%CI 0.1 to 0.2, *p* < .001, respectively) and in TREHIIT (0.9 mmol/mol, 95%CI 0.2 to 1.6, *p* = .009, and 0.1, 95%CI 0.1 to 0.2, *p* < .001, respectively), while fasting glucose increased in CON (0.3 mmol/L, 95%CI 0.1 to 0.6, *p* = .004) (Supplementary Table S2, Fig. 5). Participants in HIIT and TREHIIT had increased VO_2_peak (2.7 mL/min/kg, 95%CI 1.0 to 4.5, *p* = .002, and 2.2 mL/min/kg, 95%CI 0.8 to 3.7, *p* = .003, respectively) after 2 years compared with baseline, while TRE had 0.2 L/min lower absolute VO_2_peak after 2 years compared with both baseline and post-intervention (Supplementary Table S2, Fig. 6). Self-reported physical activity and sleep quality scores did not differ between groups (Table 2), while sleep quality improved in TREHIIT after 2 years compared with baseline (Supplementary Table S2).

**Table 2:** Linear mixed model intention-to-treat analyses of cardiometabolic outcomes and subjective physical activity and sleep. Descriptive data at baseline, after the 7-week intervention, and after 2 years for *n* participants in each group. Baseline data and 7-week data are from all participants included in the TREHIIT trial, while data after 2 years are from all participants attending the 2-year follow-up. The difference (group X time) is the mean change from baseline to 2 years in the intervention groups compared with the control group (CON). *p* values <. 01 are considered statistically significant.

	Baseline		After the 7-week intervention		After 2 years		Between-group difference after 2 years		
	*n*	**Mean (SD)**	*n*	**Mean (SD)**	*n*	**Mean (SD)**	**Estimate**	**95% CI**	***p***
**Body mass**,** kg**									
CON	33	95.0 (11.2)	29	94.2 (12.0)	10	93.9 (11.1)			
TRE	33	91.0 (10.8)	29	89.4 (12.3)	15	85.2 (9.6)	−1.1	−4.4 to 2.2	0.517
HIIT	33	91.3 (13.0)	26	90.4 (13.8)	12	85.1 (13.4)	−4.2	−7.7 to −0.7	0.019
TREHIIT	32	88.2 (10.3)	27	84.9 (10.6)	19	82.6 (11.1)	−2.7	−5.9 to 0.1	0.104
**Fat mass**,** kg**									
CON	33	39.5 (10.1)	29	38.4 (10.4)	10	37.8 (8.8)			
TRE	33	37.3 (7.6)	29	35.9 (8.8)	14	34.4 (6.8)	−0.8	−3.7 to 2.1	0.566
HIIT	33	38.6 (10.5)	26	37.7 (11.7)	12	34.9 (11.9)	−3.1	−6.1 to −0.1	0.041
TREHIIT	32	35.8 (8.4)	27	33.2 (8.9)	19	31.4 (9.8)	−2.8	−5.6 to −0.1	0.043
**Muscle mass**,** kg**									
CON	33	31.0 (3.1)	29	31.1 (3.2)	10	31.3 (3.2)			
TRE	33	30.0 (2.9)	29	29.7 (3.0)	14	28.6 (2.5)	−0.2	−0.8 to 0.4	0.563
HIIT	33	29.3 (2.4)	26	29.4 (2.3)	12	27.8 (2.5)	−0.6	−1.2 to 0.1	0.092
TREHIIT	32	29.2 (2.6)	27	28.7 (2.6)	19	28.5 (2.3)	0.3	−0.3 to 0.8	0.390
**Visceral fat area**,** cm**^**2**^									
CON	33	187 (49)	29	182 (50)	10	180 (44)			
TRE	33	180 (35)	29	173 (42)	14	168 (35)	−6	−22 to 9	0.416
HIIT	33	185 (48)	26	177 (54)	12	167 (58)	−15	−31 to 0	0.055
TREHIIT	32	172 (38)	27	159 (43)	19	148 (47)	−18	−33 to −4	0.015
**Fasting glucose**,** mmol/L**									
CON	32	5.0 (0.4)	28	5.0 (0.4)	10	5.4 (0.3)			
TRE	33	5.0 (0.5)	28	4.9 (0.5)	13	5.2 (0.6)	−0.1	−0.4 to 0.2	0.476
HIIT	33	4.9 (0.4)	26	4.8 (0.5)	12	4.9 (0.2)	−0.3	−0.6 to −0.0	0.040
TREHIIT	32	4.9 (0.4)	27	4.8 (0.4)	19	5.0 (0.4)	−0.3	−0.6 to 0.0	0.052
**HbA1c**,** mmol/mol**									
CON	31	33.8 (3.0)	27	34.2 (2.8)	10	34.3 (2.8)			
TRE	32	34.5 (2.9)	26	34.7 (3.1)	13	34.9 (3.0)	−0.6	−1.8 to 0.5	0.299
HIIT	33	33.1 (3.5)	26	32.9 (4.0)	12	35.2 (3.0)	0.1	−1.1 to 1.3	0.901
TREHIIT	30	34.6 (3.6)	26	33.9 (3.5)	19	34.4 (3.5)	−0.8	−1.9 to 0.3	0.144
**Total cholesterol**,** mmol/L**									
CON	31	4.8 (1.1)	27	4.6 (1.1)	10	4.8 (1.1)			
TRE	32	4.7 (0.7)	26	4.7 (0.6)	13	4.7 (0.6)	−0.0	−0.4 to 0.3	0.997
HIIT	33	4.8 (0.7)	26	4.5 (0.6)	12	4.9 (0.7)	−0.0	−0.4 to 0.4	0.979
TREHIIT	30	4.7 (0.7)	26	4.6 (0.7)	19	4.8 (0.7)	−0.1	−0.5 to 0.2	0.378
**HDL cholesterol**,** mmol/L**									
CON	31	1.3 (0.3)	27	1.3 (0.3)	10	1.4 (0.3)			
TRE	32	1.3 (0.3)	26	1.3 (0.2)	13	1.4 (0.3)	0.0	−0.1 to 0.1	0.635
HIIT	33	1.4 (0.3)	26	1.4 (0.3)	12	1.6 (0.3)	0.1	−0.0 to 0.2	0.148
TREHIIT	30	1.4 (0.3)	26	1.3 (0.3)	19	1.5 (0.3)	0.0	−0.1 to 0.1	0.880
**LDL cholesterol**,** mmol/L**									
CON	31	3.4 (1.1)	27	3.3 (1.1)	10	3.4 (1.0)			
TRE	32	3.2 (0.7)	26	3.3 (0.7)	13	3.2 (0.8)	−0.2	−0.5 to 0.2	0.404
HIIT	33	3.3 (0.8)	26	3.1 (0.8)	12	3.3 (0.7)	−0.1	−0.5 to 0.3	0.542
TREHIIT	30	3.3 (0.7)	26	3.2 (0.7)	19	3.4 (1.0)	−0.1	−0.5 to 0.2	0.433
**Triglycerides**,** mmol/L**									
CON	31	1.2 (0.6)	27	1.1 (0.5)	10	1.1 (0.4)			
TRE	32	1.3 (0.5)	26	1.2 (0.5)	13	1.1 (0.4)	−0.0	−0.3 o 0.3	0.914
HIIT	33	1.1 (0.4)	26	1.1 (0.4)	12	1.2 (0.7)	0.1	−0.2 to 0.4	0.340
TREHIIT	30	1.0 (0.4)	26	1.1 (0.5)	19	0.9 (0.4)	−0.1	−0.3 to 0.2	0.651
**VO**_**2**_ **peak**,** L/min**									
CON	33	3.2 (0.4)	29	3.2 (0.4)	9	3.4 (0.3)			
TRE	33	3.1 (0.3)	27	3.1 (0.4)	14	2.9 (0.4)	−0.3	−0.5 to −0.1	0.012
HIIT	33	3.1 (0.4)	26	3.3 (0.4)	12	3.1 (0.3)	−0.0	−0.2 to 0.2	0.832
TREHIIT	32	3.0 (0.4)	26	3.2 (0.4)	18	3.1 (0.4)	−0.0	−0.2 to 0.2	0.625
**VO**_**2**_ **peak**,** mL/min/kg**									
CON	33	34.6 (5.7)	29	34.6 (6.0)	9	36.8 (4.3)			
TRE	33	35.0 (5.0)	27	35.3 (5.3)	14	35.3 (4.1)	−1.5	−4.0 to 1.1	0.255
HIIT	33	34.6 (6.1)	26	36.8 (5.9)	12	36.6 (5.0)	1.6	−1.0 to 4.3	0.218
TREHIIT	32	34.8 (5.5)	26	38.1 (5.7)	18	37.9 (6.8)	1.1	−1.3 to 3.6	0.357
**Systolic BP**,** mmHg**									
CON	33	122.4 (10.3)	29	122.6 (10.5)	10	123.8 (10.9)			
TRE	33	121 (10.7)	28	118.7 (11.5)	14	117.7 (8.0)	−3.9	−9.4 to 1.7	0.169
HIIT	33	122.6 (10.1)	26	121.5 (9.3)	11	116.6 (9.8)	−2.9	−8.8 to 2.9	0.324
TREHIIT	32	124.5 (10.9)	26	124.7 (8.2)	19	119.4 (7.3)	−1.7	−7.0 to 3.6	0.522
**Diastolic BP**,** mmHg**									
CON	33	80.4 (8.4)	29	80.3 (9.9)	10	82.8 (9.4)			
TRE	33	79.9 (9.0)	28	78.6 (10.1)	14	78.7 (6.2)	−4.3	−8.9 to 0.4	0.071
HIIT	33	78.8 (7.9)	26	78.3 (8.0)	11	78.0 (6.0)	−1.7	−6.6 to 3.1	0.482
TREHIIT	32	82.6 (8.3)	26	81.5 (5.7)	19	79.5 (5.5)	−3.5	−7.9 to 0.9	0.118
**Resting heart rate**,** bpm**									
CON	33	71.0 (9.1)	29	71.2 (10.3)	9	66.8 (4.6)			
TRE	33	70.3 (8.1)	28	71.2 (9.2)	14	67.5 (6.4)	0.8	−5.5 to 7.1	0.801
HIIT	33	71.9 (9.5)	26	68.0 (9.5)	12	71.4 (8.8)	4.7	−2.0 to 11.3	0.168
TREHIIT	32	69.9 (11.5)	26	67.5 (11.8)	19	68.2 (7.9)	1.9	−4.1 to 7.9	0.527
**IPAQ score**									
CON	29	2449 (2445)	29	2195 (2561)	11	2494 (1504)			
TRE	30	1489 (1411)	27	1718 (2401)	16	1524 (1151)	−837	−2103 to 429	0.194
HIIT	29	1247 (1157)	26	1582 (1182)	12	1666 (1434)	−596	−1940 to 748	0.383
TREHIIT	29	1697 (2430)	27	2767 (2493)	20	2549 (2723)	61	−1156 to 1277	0.922
**PSQI score**									
CON	28	5.9 (3.9)	28	4.8 (3.5)	10	5.8 (4.1)			
TRE	29	6.8 (2.9)	27	5.5 (2.5)	15	5.1 (2.6)	0.1	−1.8 to 2.0	0.898
HIIT	30	6.4 (3.3)	26	5.7 (2.5)	12	5.6 (3.6)	0.7	−1.3 to 2.6	0.499
TREHIIT	30	6.6 (3.9)	28	5.5 (3.1)	20	4.5 (2.3)	−0.7	−2.4 to 1.1	0.482

*BP* blood pressure, *CON* control, *HbA1c* glycated haemoglobin, *HDL* high-density lipoprotein, *HIIT* high-intensity interval training, *IPAQ* International physical activity questionnaire, *LDL* low-density lipoprotein, *PSQI* Pittsburgh sleep quality index, *TRE* time-restricted eating, *TREHIIT* time-restricted eating and high-intensity interval training, *VO*_*2*_*peak* peak oxygen uptake.

## Discussion

We investigated self-reported continuation of TRE and HIIT among women with overweight/obesity two years after completing a 7-week RCT. Although participants were not encouraged to continue the interventions beyond trial completion, nearly half of the participants reported that they engaged in TRE and/or HIIT during the follow-up period. Specifically, 46% of participants in the follow-up study who had undergone the TRE intervention in the RCT or as delayed treatment (for participants in the original CON group), reported maintaining a ≤ 10-h TRE window on approximately 5 days/week. Similarly, 45% of participants who received the HIIT intervention in the RCT or as delayed treatment reported engaging in HIIT 1–2 times/week after 2 years. We found no significant differences between the initial study groups in cardiometabolic outcomes after 2 years, but there were tendencies of improved body composition among those initially allocated to the HIIT or TREHIIT groups, compared with CON. We did not observe any further reductions in body weight across any of the groups after 2 years, but participants in HIIT and TREHIIT had significantly lower fat mass and visceral fat area compared with baseline.

The continuation rates of TRE in our study were similar to a previous study in which participants at risk of type 2 diabetes reported to consume all energy within a 10-h TRE window on 45% of the days during a 3-month follow-up period without active intervention^34^. During the active intervention, however, the participants consumed all energy withing the 10-h window on 91% of the days^34^. In contrast, others have reported that 63% of participants with metabolic syndrome adhered to an 8–12-h TRE window ~ 16 months after completing a 12-week intervention^35^. Differences in methods of collecting adherence data might partly explain these variations, as Quist et al.^34^ collected weekly adherence data via an online diary, likely providing more accurate results, while both Wilkinson et al.^35^ and we relied on retrospective self-reported data. Recall bias may have influenced our findings, limiting their reflection of TRE maintenance during the entire 2-year follow-up period. Future long-term studies could benefit from continuous monitoring of adherence to better understand how TRE is maintained in the real-world.

Self-reported continuation of HIIT in our study (45%) was slightly higher than in a 1-year study of unsupervised HIIT, in which 39% of adults with overweight/obesity completed at least one HIIT session/week after one year^36^. In another study, 59% of participants with overweight/obesity who completed a fully supervised 8-week HIIT intervention reported exercising regularly during a 4-month follow-up period, with no significant changes in moderate-to-vigorous physical activity measured with accelerometer^37^. In that study, the participants were explicitly encouraged to continue exercising, which may, in addition to the shorter follow-up period explain the higher adherence rates than in our study. Although methodological differences make comparisons between studies challenging, declining HIIT adherence over time is well-documented^25,38–40^.

Despite initial reductions in fat mass and visceral fat area after 7 weeks of the TRE, HIIT, and TREHIIT interventions compared with CON, no significant between-group differences were observed after 2 years. Our results are likely influenced by both discontinuation of TRE and/or HIIT and intervention “crossover” post-trial. Most of the participants from the CON group completed a 7-week delayed treatment of HIIT or TREHIIT immediately after the control period. Additionally, four participants in the TRE group reported starting with HIIT after study completion, while three in the HIIT group reported starting with TRE.

Most participants across all groups in our study reported that they started with various exercise forms after study completion. Regular exercise is associated with better weight-loss maintenance^17,18^. However, only the HIIT and TREHIIT groups maintained lower body mass, fat mass, and visceral fat area after 2 years compared with baseline. Additionally, only HIIT and TREHIIT maintained improvements in VO_2_peak, while the TRE group had reduced absolute VO_2_peak after 2 years compared with baseline. The initial intervention of supervised HIIT may have induced greater self-efficacy^41,42^, and increased the likelihood of regularly engaging in high-intensity exercise after the trial, possibly contributing to sustained fitness. However, there seemed to be a shift towards lower-intensity exercise in all groups, which could explain the lack of any further improvements in body composition and cardiorespiratory fitness from post-intervention to the 2-year follow-up.

Twenty-one participants reported taking up resistance training after trial completion, and 11 reported engaging in moderate-intensity endurance exercise. Findings from previous long-term studies of HIIT also show declining rates of completed HIIT sessions and increased engagement in moderate-intensity physical activity^42^. Participants with overweight or obesity who were prescribed three weekly HIIT sessions in free-living conditions for 12 months reported a decline in weekly HIIT sessions from 1.9 ± 0.9 to 1.0 ± 0.9 after 12 months, and a concomitant increase in moderate-intensity exercise^39^. Similarly, the participants in our study who still engaged in HIIT after 2 years also reported completing 1–2 sessions weekly. Maintaining high levels of vigorous activity without supervision in the long-term seems to be challenging. Notably, the problems with long-term adherence do not differ between HIIT and moderate-intensity exercise^42^, and efforts need to be made to improve long-term maintenance of any exercise form.

The participants in our study generally expressed positive attitudes towards HIIT, rating it favourable compared with other exercise strategies. Positive affective responses during exercise can predict future participation in physical activity^43^, and current evidence show similar or greater post-exercise affective responses after completed HIIT sessions compared with moderate-intensity continuous exercise^42^. Although HIIT can elicit exercise enjoyment in populations with overweight or obesity^44–46^, long-term adoption of any exercise does not rely solely on its immediate affective response. Indeed, mixed perceptions towards HIIT were revealed in our participants’ qualitative responses. The most stated reason for not adopting HIIT in our study was lack of time, which is a common perceived barrier to regular exercise^47^. Simultaneously, “time-efficiency” was the most frequently stated positive aspect of HIIT. Indeed, HIIT is often referred to as a time-efficient alternative to the traditional recommendations of 150 min/week of moderate-intensity continuous training^39,48^.

The nature of HIIT, with alternating short bursts of vigorous activity and lower intensity recovery periods, is suggested to induce emotional experiences such as a sense of pride and accomplishment after completing each high-intensity bout^48–50^. Indeed, one participant specifically mentioned that the variations in intensity within the session made the time pass faster, and six participants reported proudness and feelings of mastery as positive attributes of HIIT. In contrast, four participants expressed aversion to HIIT due to the high physical exertion required, supporting an exercise intensity-affect relationship^51^. These mixed perceptions highlight the need for personalized approaches in sustainable exercise strategies.

TRE was also favourably rated by our participants, compared with other diet strategies they had attempted. TRE was consistently perceived as easy to follow, aligning with previous reports^16,52^. However, challenges with TRE adherence are reported to arise if substantial adjustments to daily activities are required^14^. While shorter and earlier TRE windows offer greater weight loss and cardiometabolic benefits, they are often less compatible with everyday life schedules, limiting long-term sustainability^53–55^. A 10-h TRE window is suggested as ideal, yielding multiple health benefits while permitting a reasonable timeframe for energy consumption^8^.

Despite prescribing a 10-h eating window in our TREHIIT trial, social events and family schedules were the most reported barriers to TRE after 2 years. Some participants who still did TRE after 2 years reported extended eating windows on the weekends. Lapses from TRE are commonly due to social happenings^14,16,52,56,57^, with non-adherence often occurring on the weekends^52,56^. Practicing a flexible approach to TRE can facilitate adherence^15,57^, and some studies show that even 5–6 days/week of TRE can have beneficial health effects^10^. Adults with obesity who reported adherence to TRE on 2.5 days in a 4-day food record had reduced adiposity after 10 weeks^57^, and adherence to 8-h TRE on 5.6 days/week over 8 weeks in healthy adults reduced body mass and systolic blood pressure^58^. It is important to consider how various adjustments to the TRE protocol interfere with health outcomes. Too much flexibility may compromise treatment effectiveness^16^, and unsatisfactory results predict attrition of lifestyle interventions^59^. Indeed, some of the participants in our study reported disappointing weight-loss outcomes as a reason for not continuing TRE. Previous research in healthy adults proposes that eating time-restricted on minimum 70% of days is necessary for acquiring substantial health benefits^60^.

There are several limitations to our study. Only a subset of the total study population in the TREHIIT trial completed the 2-year follow-up, introducing self-selection bias and limiting statistical power to detect differences in outcomes. There is a risk of type 1 error due to multiple comparisons, despite considering *p*-values < 0.01 as statistically significant. Self-reported continuation of diet and exercise is prone to recall bias, and the reported continuation of TRE and HIIT might not correspond with overall engagement throughout the entire follow-up period. The lack of objective physical activity data and collection of adherence data at interim time-points during the follow-up period also limits our ability to draw conclusions on whether the amount of high-intensity exercise could explain our findings of maintained cardiorespiratory fitness in the HIIT and TREHIIT groups.

## Conclusions

In this follow-up study, almost half of the included participants reported undertaking some amount of TRE and/or HIIT 2 years after completing a 7-week intervention period. Participants originally allocated to HIIT and TREHIIT maintained improvements in body composition and cardiorespiratory fitness. Despite reduced adherence to the initial interventions, an intensive TRE and HIIT intervention may have long-lasting effects on lifestyle behaviour for sustained health benefits. Overall, TRE and HIIT were regarded as attractive diet-exercise alternatives, but personalized and flexible approaches to TRE and HIIT are likely needed to enhance long-term adoption.

## Electronic supplementary material

Below is the link to the electronic supplementary material.

Supplementary Material 1

Supplementary Material 2

Supplementary Material 3

## Data Availability

Deidentified participant data underlying the results in this article is available from the corresponding author upon reasonable request.

## Abbreviations

CER: continuous energy restriction
CON: control
HbA1c: glycated haemoglobin
HDL: high-density lipoprotein
HIIT: high-intensity interval training
LDL: low-density lipoprotein
TRE: time-restricted eating
TREHIIT: time-restricted eating and high-intensity interval training
VO_2_peak: peak oxygen uptake
