# Supplementary material for: Maintenance of time-restricted eating and high-intensity interval training in women with overweight/obesity 2 years after a randomized controlled trial
Source: Sci Rep. 2025 Apr 25;15:14520. doi: 10.1038/s41598-025-95743-y (PMC12032017; doi:10.1038/s41598-025-95743-y)
Supplement: Supplementary file 2 — Supplementary Material 2 [file 41598_2025_95743_MOESM2_ESM.pdf]

Maintenance of time-restricted eating and high-intensity interval training in women with overweight/obesity 2 years after a randomized controlled trial

Kamilla L. Haganes, John A. Hawley, Stian Lydersen, Trine Moholdt

Supplementary information

Table S1

Table S2 (Separate excel file)

Long-term adherence and perceptions questionnaire

Table S1. Qualitative questionnaire responses on perceptions of time-restricted eating (TRE) and high-intensity interval training (HIIT) by n = 10 in TRE, n = 9 in HIIT, n = 19 in TREHIIT, n = 6 in CON.

**Perceptions of TRE**	**Group**
Quote	
I have no problem not eating dinner. [I] became energetic, [and] also gained a stronger sense of self-esteem by doing so.	TRE
Difficult to compare, [I] have only changed dietary quality.	TRE
Works well to have focus and consciousness around food.	TRE
Almost never hungry in the morning anyway, a small sacrifice to give up dinner. Free to eat anything.	TRE
Can eat whatever you want, but [I'm] still a bit hungry.	TRE
It's okay to follow but hasn't yielded results.	TRE
For the most part okay to follow over time.	TRE
Quite easy to follow.	TRE
Haven't tried any other [diets].	TRE
Simple rules, easy to understand what is required.	TRE
A good combo of diet plan and time-restricted eating.	TREHIIT
Simple and ok to follow. Avoids snacking in the evening.	TREHIIT
Don't like eating small meals frequently, [I] prefer two large meals instead.	TREHIIT
Not very demanding, but little impact and not very motivating.	TREHIIT
Easy to understand and follow.	TREHIIT
A relief not having to eat breakfast. Saves time.	TREHIIT
Easier to do once you get into the routine; when the cravings in the evenings are gone, it's easy.	TREHIIT
Quickly noticed stabilization of blood sugar throughout the day. Avoiding blood sugar peaks and lows. Don't feel hunger as intensely.	TREHIIT
Easier to follow! Easier to be social.	TREHIIT
Easy to understand and follow.	TREHIIT
Easy to follow.	TREHIIT
It's simple. Watching the time.	TREHIIT
I find that it was easier to engage in fasting during the study due to little change in daily routines.	TREHIIT
Easy to follow.	TREHIIT
Easy to stick to the plan, better sleep, more consistent with water intake.	TREHIIT
Doesn't impose restrictions on WHAT you eat, especially if you're invited somewhere, it's much easier to adapt.	TREHIIT
Doesn't require much preparation or special acquirements. Just need to watch the timing.	TREHIIT
Don't know.	CON
Don't need to think about what or how much you eat, only when.	CON
Much easier to maintain, no need for specific foods, requires no time commitment.	CON
Cravings and hunger disappear quickly, and after a certain point, the weight drops rapidly without much effort.	CON
**Perceptions of HIIT**	**Group**
Quote	
I enjoy [HIIT], but I also like other types [of exercise] (e.g., strength training, hiking).	HIIT
Terrible to run and feel like you can't breathe. Claustrophobic.	HIIT

Not as fun to do, it's heavier.	HIIT
I see that if I do interval training, it's better in terms of weight reduction.	HIIT
Doesn't take too long. Fun if the fitness level is okay.	HIIT
It doesn't take too long, and the variations [within the sessions] make the time pass faster.	HIIT
Tiring, but I see progress, fun.	HIIT
Feel like I master it. Good feeling in the body afterwards.	HIIT
[I] like feeling the heart pumping.	HIIT
A bit easier on the back and less muscle soreness.	TREHIIT
Just a short half-hour, and you're done.	TREHIIT
Time-efficient and easy to perform without equipment.	TREHIIT
Effective when you're short on time.	TREHIIT
Efficient. Rapid progression. Sense of mastery. Achieved more than I thought. Overcame barrier to outdoor jogging in sleet.	TREHIIT
Both [resistance training and HIIT] require a discipline that I find difficult to muster.	TREHIIT
Intense and effective, I like it a lot.	TREHIIT
Weight makes everything heavy; it needs to be combined with everyday life and the remaining energy and desire after work. Sometimes, it's physically better to take a 60-minute walk than endure 25 minutes of intense effort.	TREHIIT
I like that you quickly notice improvement in endurance.	TREHIIT
It's manageable. Provides good results with a short training time.	TREHIIT
Efficient. Both timesaving and yield results rapidly.	TREHIIT
Efficient, takes little time.	TREHIIT
Quick and effective.	TREHIIT
Yields results with minimal time-investment but requires significant discipline to endure the discomfort during high percentage of maximal heart rate.	TREHIIT
Simple exercise, but heavy to start with after a break [from training].	TREHIIT
Effective.	TREHIIT
Effective, timesaving, pushes me to the limit, and I feel proud of it.	TREHIIT
Efficient, [you] get a lot of training done in a short amount of time. A good way to ‘brain dump’.	TREHIIT
Time-efficient, don't need to plan much.	TREHIIT
Short, effective sessions that yield quick results.	CON
Efficient to carry out. Good effect in a short time.	CON
Effective and fun.	CON
Much better, [I] feel [I run] faster and in better shape, but it makes the next workout a bit challenging to approach as I dread the high level of exertion.	CON
Effective.	CON
Effective, but it can become monotonous if practiced all the time.	CON

CON control group, *HIIT* high-intensity interval training*, TRE* time-restricted eating*, TREHIIT* time-restricted eating and high-intensity interval training. Note: The authors have directly translated the quotes from Norwegian to English.

Long-term adherence and perceptions questionnaire

TREHIT- A follow-up study   ID-number ☐☐☐

We ask you to fill out this questionnaire. Your answers are an important contribution to understand the adherence to time-restricted eating and/or high-intensity interval training after participating in a randomized controlled trial.

Please answer all the questions. Do not spend too long on each question. The first thing that comes to your mind is usually the correct answer.

Your answer must appear clearly. Use black or dark blue pen for filling. If you answer incorrectly, completely fill the square and check the correct box.

Today's date *(ddmmyyyy)*: ☐☐·☐☐·☐☐☐☐

INITIAL GROUP

1) Which group were you allocated to in the main study?

☐ Time-restricted eating (Proceed to page 2)

☐ High-intensity interval training (Proceed to page 4)

☐ Time-restricted eating and High-intensity interval training (Fill out page 2-5)

☐ Control (Proceed to question 2)

2) If you were in the control group, which group did you choose after post-tests?

☐ Time-restricted eating (Proceed to page 2)

☐ High-intensity interval training (Proceed to page 3)

☐ Time-restricted eating and High-intensity interval training (Fill out page 2-3)

☐ No group (Proceed to page 6)

TIME-RESTRICTED EATING

3) Have you continued with time-restricted eating (fully/partly) after study completion?

☐ YES ☐ NO

If YES

a) **For how long did you continue with time-restricted eating?**

	Number of
☐ Some weeks	☐☐ weeks
☐ Some months	☐☐ months
☐ Still doing it	

b) **How long is/was your eating window on the weekdays?**

☐☐ hours/day Time window (timepoints): First meal: ☐☐:☐☐

Last meal: ☐☐:☐☐

c) **How long is/was your eating window on the weekends?**

☐☐ hours/day Time window (timepoints): First meal: ☐☐:☐☐

Last meal: ☐☐:☐☐

d) **How many days per week have you done time-restricted eating (on average)?**

☐ days per week

If NO

e) **What was the reason(s) for not continuing with time-restricted eating? You can check as many boxes as applies to you**

☐ Did not align with work-schedule

☐ Family routines

☐ Didn't get the results I wanted

☐ Difficult to maintain in social situations

☐ Other reasons:____

4) Have you tried any other diet strategies before and/or after participating in the main study?

- ☐ No
- ☐ 5:2 Periodic fasting
- ☐ Low carb
- ☐ Calorie counting
- ☐ Grethe Roede
- ☐ Other: ____

a) **How would you rate time-restricted eating compared with other diet strategies you have tried? (Mark with a cross)**

1	2	3	4	5	6	7	8	9	10

Much worse — Neither better nor worse — Much better

Reason:____

____

5) Have you started with any exercise after study completion?

- ☐ No
- ☐ Resistance training
- ☐ High-intensity interval training
- ☐ Group exercise at a gym
- ☐ Moderate-intensity continuous exercise
- ☐ Other: __________________________________

____

HIGH-INTENSITY INTERVAL TRAINING

6) Have you continued with high-intensity interval training (fully/partly) after study completion?

☐ YES ☐ NO

If YES

a) For how long did you continue with high-intensity interval training?

	Number of
☐ Some weeks	☐☐ weeks
☐ Some months	☐☐ months
☐ Still doing it	

b) How many days per week do you complete high-intensity interval training?

☐ days per week

c) What type of high-intensity interval training have you completed?

☐ 4 x 4

☐ 10 x 1

☐ Both

☐ Other: ____

If NO

d) What was the reason(s) for not continuing with high-intensity interval training? You can check as many boxes as applies to you

☐ Too hard

☐ Lack of time

☐ Boring

☐ Did not get the results I wanted

☐ Other ____

7) Have you started with any other form of exercise after study completion?

- ☐ No
- ☐ Group exercise at a gym
- ☐ Moderate-intensity continuous exercise
- ☐ Other: ______________________________
- ☐ Resistance training

a) **How would you rate high-intensity interval training compared with other exercise strategies you have tried? (Mark with a cross)**

1	2	3	4	5	6	7	8	9	10

Much worse — Neither better nor worse — Much better

Reason: ____

8) Have you started with any dietary strategies after study completion?

- ☐ No
- ☐ 5:2 Periodic fasting
- ☐ Time-restricted eating
- ☐ Calorie counting
- ☐ Low carb
- ☐ Grethe Roede
- ☐ Other: ______________________________

CONTROL GROUP

9) What was the reason(s) for not chosing a delayed intervention after study completion?

____

____

____

10) Have you started with any dietary strategies after study completion?

- ☐ No
- ☐ 5:2 Periodic fasting
- ☐ Time-restricted eating
- ☐ Calorie counting
- ☐ Low carb
- ☐ Grethe Roede
- ☐ Other: ____

11) Have you started with any other form of exercise after study completion?

- ☐ No
- ☐ Group exercise at a gym
- ☐ Moderate-intensity continuous exercise
- ☐ High-intensity interval training
- ☐ Resistance training
- ☐ Other: ____________________________
